# Malignancy and IgG4-related disease: the incidence, related factors and prognosis from a prospective cohort study in China

**DOI:** 10.1038/s41598-020-61585-z

**Published:** 2020-03-18

**Authors:** Hanqi Tang, Huaxia Yang, Panpan Zhang, Di Wu, Shangzhu Zhang, Jiuliang Zhao, Linyi Peng, Hua Chen, Yunyun Fei, Xuan Zhang, Yan Zhao, Xiaofeng Zeng, Fengchun Zhang, Wen Zhang

**Affiliations:** 1 Department of Rheumatology and Clinical Immunology, Peking Union Medical College Hospital, Chinese Academy of Medical Sciences and Peking Union Medical College, The Ministry of Education Key Laboratory, Beijing, 100730 China; 2 National Clinical Research Center for Dermatologic and Immunologic Diseases, Beijing, China

**Keywords:** Cancer epidemiology, Connective tissue diseases, Risk factors

## Abstract

This prospective cohort study aims to investigate the incidence, related factors and prognosis of IgG4-related disease (IgG4-RD) with malignancies in the Chinese cohort. We prospectively analyzed the IgG4-RD patients recruited in Peking Union Medical College Hospital from January 2011 to August 2018 and identified patients diagnosed with IgG4-RD complicating malignancies. Data regarding demographics, clinical features, treatment and prognosis of IgG4-RD patients complicating malignancies were collected and compared to those of age- and sex-matched controls. Among the 587 Chinese patients with IgG4-RD, 17 malignancies were identified. Ten of them developed malignancy after the diagnosis of IgG4-RD, given a standard incidence ratio (SIR) of 2.78 (95%CI 1.33–5.12). Multivariate logistic analysis indicated that autoimmune pancreatitis (OR = 6.230, 95%CI 1.559–24.907, p = 0.010) was positively associated with malignancy, whereas eosinophilia (OR = 0.094, 95%CI 0.010–0.883, p = 0.039) was negatively related with malignancies. During a median follow-up period of 61.4 ± 26.4 months, all patients with IgG4-RD and malignancies survived. We conclude that an increased incidence of malignancy was found in Chinese IgG4-RD cohort. Autoimmune pancreatitis is a potential risk factor, whereas eosinophilia is a possible protective factor for complicating malignancies.

## Introduction

ImmunoglobulinG4-related disease (IgG4-RD) is an immune-mediated fibroinflammatory condition that is typically diagnosed by clinicopathological presentations of enlarged organs, elevation of serum concentrations of IgG4 and pathological findings such as a typical dense lymphoplasmacytic infiltration enriched in IgG4-positive plasma cells, storiform fibrosis, and obliterative phlebitis^1,2^. Their mass-like clinicopathological presentations and radiologically ^18^F-FDG-avid hypermetabolic lesions has made it to be one of the great malignancy mimickers^2^. Previous studies have reported that a spectrum of malignancies occur in association with IgG4-RD, including solid tumors located in the lung, pancreas, gastrointestinal tract, and prostate, as well as lymphoma^3–10^. Moreover, it is difficult for clinicians to make a diagnosis of IgG4-RD because it can manifest as mass lesions, leading to an occasional misdiagnosis of malignancy. The relationships between these two diseases remain unclear and are the subject of on-going research. However, the mechanism underlying the co-occurrence of IgG4-RD and malignancies has not been fully determined to date.

Wallace reported an increased standardized prevalence ration of malignancy history in IgG4-RD patients^5^, while studies from Asia, Europe and America have also suggested an increased standardized incidence ratio of malignancy in patients with IgG4-RD^3,4,6,7,9,10^. However, one study from Japan reported no significant increase in the risk of malignancy^8^. No epidemiological data for malignancies in Chinese IgG4-RD patients is available. It is also important to know the risk factors and prognosis for IgG4-RD patients harboring malignancies.

Our team at Peking Union Medical College Hospital (PUMCH) established an IgG4-RD cohort in 2011. In previous studies, we prospectively described the clinical characteristics^11^, sex disparities^12^, radiographic presentation^13^ and histopathologic features^14^ of patients with IgG4-RD in our cohort. The present study focused on the association between IgG4-RD and malignancies. We sought to describe the incidence and clinical characteristics of malignancies in patients with IgG4-RD. Based on these results, significant factors were evaluated to determine whether they were related to malignancy. Treatments and survivals were also generated to characterize the prognosis.

## Methods

### Patients and controls

We consecutively collected data on patients with IgG4-RD and malignancies who were prospectively enrolled in the IgG4-RD cohort in PUMCH from January 2011 and June 2018. IgG4-RD was diagnosed according to the 2011 comprehensive diagnostic criteria for IgG4-RD^1^. Malignancies were diagnosed histopathologically by biopsy or resected specimens according to the International Classification of Disease (ICD-10) criteria. Patients with IgG4-RD and malignancy diagnosed at the same time were defined as malignancy diagnosis within 1 year’s duration of IgG4-RD diagnosis. Patients with malignancy history or newly diagnosed malignancy were defined as >1 year before or after the diagnosis of IgG4-RD. To identify the risk factors for malignancies in patients with IgG4-RD, We randomly selected sex and age match IgG4-RD patients without malignancies as controls at a ratio of 3:1 from our cohort. This study was approved by the Medical Ethics Committee of Peking Union Medical College Hospital (No. S-K412) and was complied with all ethics committee requirements. Written informed consent were obtained from all patients and/or their legal guardians.

### Data collection

Baseline data concerning demographics, past medical and personal histories, organ involvement and laboratory findings were recorded. Past medical histories included allergy, smoking histories. Laboratory findings were obtained from individual patients when they first diagnosed with IgG4-RD at our institution. Treatments for both IgG4-RD and malignancies were documented. All patients recruited in our cohort were consecutively followed-up in our rheumatology clinic every 3 to 6 months. Death and causes of death were determined by direct contact with the attending clinician or reported by telephone followed-up.

### Statistical analysis

The standardized incidence rate (SIR) was calculated by dividing the observed newly developed malignant rate by the expected rate. Expected malignancies were calculated by age-stratified malignancy incidence (cases per 100000 person-year) in general Chinese population multiplying the corresponding number of patients in each age-group. The data of sex- and age- stratified cancer incidence in general population were published before by the Chinese National Cancer Center^15,16^. Continuous variables were summarized as the means ± standard deviation (SD) or medians and interquartile range (IQR), and categorical data were presented as frequencies (percentages). The Wilcoxon rank sum test and chi-squared tests for independence were utilized to evaluate significant differences in continuous data and categorical data, respectively. To identify risk factors for malignancy in IgG4-RD patients, univariate and stepwise multivariate logistic regression analyses were performed. All statistical analyses were performed with SPSS software (version 23.0).

## Results

### Demographics of IgG4-RD patients complicating with malignancy

**Figure 1:**
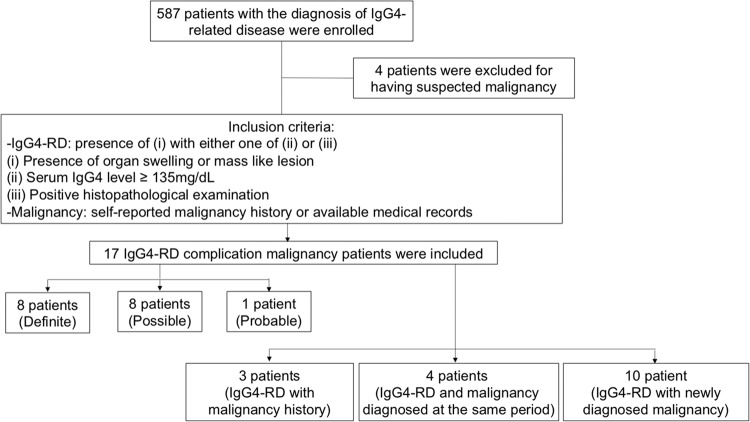
Flowchart of inclusion of patients with IgG4-RD and malignancy.

A total of 587 IgG4-RD patients in the PUMCH cohort were consecutively enrolled in this study. Seventeen patients were identified as having malignancies (Table 1, Fig. 1). Among these IgG4-RD with malignancy patients, 11 (64.7%) were male and 6 (35.3%) were female, with mean disease duration of 61.4 ± 26.4 months (Table 2). The mean ages were 56.8 ± 13.0 years at the diagnosis of IgG4-RD and 57.5 ± 12.7 years at the diagnosis of malignancy.

**Table 1:** Baseline characteristic of IgG4-RD patients with malignancy.

Patient	Sex	Age	Age at diagnosis of IgG4-RD	Age at diagnosis of malignancy	Serum IgG4 at diagnosis of IgG4-RD (g/L)	Organs involvements of IgG4-RD (*Organ with biopsy)	Sites of malignancy
P1	F	59	58	54	1499	Parotid gland*, salivary gland	Breast cancer
P2	M	74	66	68	10402	Pancreas, bile duct, retroperitoneal fibrosis, kidney, prostate, lymph nodes	Rectal cancer
P3	M	46	42	40	2630	Lacrimal gland, parotid gland	Lipoblastoma
P4	M	70	68	64	5780	Pancreas, bile duct, lung, prostate, lymph nodes	Thyroid carcinoma
P5	F	62	61	61	11600	Pancreas, bile duct, salivary gland*, periaortitis, lymph nodes, pituitary	Thyroid carcinoma
P6	M	72	68	68	3490	Pancreas, bile duct, lymph nodes	Rectal cancer
P7	M	60	58	58	2410	Pancreas, bile duct, lymph nodes	Renal cancer
P8	M	68	63	68	3520	Pancreas, bile duct, retroperitoneal fibrosis, lung, kidney, artery, lymph nodes	Rectal cancer
P9	M	36	30	35	12400	Pancreas, bile duct	Skin cancer
P10	M	52	49	52	10000	Pancreas, parotid gland*, lacrimal gland, lung, prostate, lymph nodes	Thyroid carcinoma
P11	F	70	68	69	17300	Parotid gland, lacrimal gland, salivary gland, sinus	Lung cancer
P12	M	82	79	79	58000	Pancreas, lacrimal gland*	Colon cancer
P13	F	50	49	45	14300	Uterus*, ovary	Ovarian carcinoma
P14	F	52	46	50	10000	Parotid gland*, lacrimal gland	Breast cancer
P15	F	60	55	57	12500	Pancreas, parotid gland, lacrimal gland, lymph nodes, sinus	Lymphoma
P16	M	42	37	40	7490	Lung*, lymph nodes	Renal cancer
P17	M	71	68	69	415	Pancreas*, bile duct	Prostate cancer

**Table 2:** Comparisons of baseline demographics, clinical features and laboratory findings in IgG4-RD patients with and without malignancy.

	Patients (n = 17)	Controls (n = 51)	p-value
**Demographic characteristics**			
Male/Female	11/6	33/18	1.000
Age at IgG4-RD diagnosis (mean ± SD, years)	56.8 ± 13.0	55.9 ± 12.2	0.707
Age at malignancy diagnosis (mean ± SD, years)	57.5 ± 12.7	NA	/
Disease duration (mean ± SD, months)	61.4 ± 26.4	75.0 ± 45.3	0.395
**Past medical history**			
Allergy, n (%)	7(41.2%)	19(37.3%)	0.778
Smoking, n (%)	9(52.9%)	19(37.3%)	0.150
Alcohol, n(%)	4(23.5%)	12(23.5%)	1.000
Organ involvements of IgG4-RD			
Head and neck, n (%)	8(47.1%)	38(74.5%)	0.036
Dacryoadenitis, n (%)	7(41.2%)	25(49.0%)	0.575
Sialadenitis, n (%)	7(41.2%)	29(56.9%)	0.262
Thyroiditis, n (%)	0(0.0%)	2(3.9%)	0.407
Sinusitis, n (%)	2(11.8%)	13(25.4%)	0.237
Gastrointestinal tract, n (%)	11(64.7%)	15(29.4%)	0.010
Sclerosing cholangitis, n (%)	7(41.2%)	10(19.6%)	0.075
Autoimmune pancreatitis, n (%)	11(64.7%)	13(25.4%)	0.003
Colitis, n (%)	0(0.0%)	2(3.9%)	0.407
Respiratory n (%)	4(23.5%)	12(23.5%)	1.000
Pulmonary involvement, n (%)	4(23.5%)	10(19.6%)	0.729
Mediastinal fibrosis, n (%)	0(0.0%)	4(7.8%)	0.234
Genitourinary n (%)	4(23.5%)	9(17.6%)	0.593
Interstitial nephritis, n (%)	2(11.8%)	5(9.8%)	0.818
Prostatitis, n (%)	3/11(27.3%)	5/33(15.2%)	0.367
Retroperitoneal fibrosis/ periaortitis, n (%)	3(17.6%)	8(15.7%)	0.849
Lymphadenopathy, n (%)	8(47.1%)	26(51.0%)	0.945
Skin involvement, n (%)	0(0.0%)	2(3.9%)	0.329
Inflammatory pseudotumour	2(11.8%)	3(5.9%)	0.421
Hypophysitis, n (%)	1(5.9%)	0(0.0%)	0.081
Baseline laboratory findings			
Eosinophilia, n (%)	1(5.9%)	16(31.4%)	0.022
Elevated ESR, n (%)	11(64.7%)	19(37.3%)	0.128
Elevated CRP, n (%)	7(41.2%)	17(33.3%)	0.627
IgG, median (IQR), g/L	18.4(14.9–21.7)	19.7(15.5–24.9)	0.581
IgA, median (IQR), g/L	2.18(1.51–3.09)	2.22(1.79–2.58)	0.831
IgM, median (IQR), g/L	0.86(0.53–1.36)	0.76(0.55–1.09)	0.670
IgE, median (IQR), mg/dL	240.5(64.0–623.5)	400(164.3–578.5)	0.374
IgG4, median (IQR), mg/dL	1000(306–1245)	552(313–1220)	0.761

IgG4-RD: IgG4-related disease; SD: standard deviation; EOS: eosinophil; ESR: erythrocyte sedimentation rate; CRP: C-reactive protein; IQR: interquartile range.

### Malignancy distribution and SIR

There were 4 (23.5%) patients reported a previous history of malignancy, and the other 13 (76.5%) patients developed malignancies at or after the diagnosis of IgG4-RD. According to the 2011 comprehensive diagnostic criteria for IgG4-RD, 8 (47.1%), 8 (47.1%) and 1 (5.8%) case were diagnosed as definite, probable and possible IgG4-RD cases, respectively (Fig. 1). One (5.9%) patient was diagnosed with lymphoma, and 16 (94.1%) patients had solid tumours. Four (23.5%) patients developed tumours in the gastrointestinal tract. The remaining solid tumours were located in the thyroid (3, 17.6%), renal (2, 11.7%), breast (2, 11.7%), and skin (1, 5.9%), lung (1, 5.9%), uterus (1, 5.9%), prostate (1, 5.9%), lipoblastoma (1, 5.9%), respectively.

**Figure 2:**
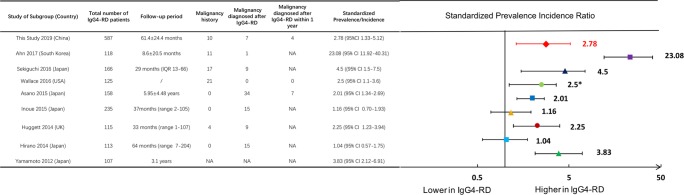
Standardized incidence ratios of malignancies in patients with IgG4-RD from different studies. US: United States; UK: United Kingdom; CI: confidence interval.

There was a significantly increased SIR of total malignancies (2.78, 95% CI 1.33–5.12) in this Chinese cohort. Figure 2 summarized the incidence ratio or prevalence ratios of malignancies in IgG4-RD patients from studies in different nations, including Japan, the UK, the US and South Korea^3–10^.

### Clinical characteristics

Baseline clinical characteristics in patients with and without malignancies were compared as shown in Table 2. IgG4-RD complicating malignancies were less likely to have head and neck involvement (47.1% versus 74.5%, p = 0.036), including dacryoadenitis (41.2% versus 49.0%, p = 0.575), sialadenitis (41.2% versus 56.9%, p = 0.262), thyroiditis (0.0% versus 3.9%, p = 0.407) and sinusitis (11.8% versus 25.4%, p = 0.237). Gastrointestinal tract involvement was found more frequently in patients with IgG4-RD complicating malignancy than in controls (64.7% versus 29.4%, p = 0.010) including sclerosing cholangitis (41.2% versus 19.6%, p = 0.075), autoimmune pancreatitis (64.7% versus 25.4%, p = 0.003), colitis (0.0% versus 3.9%, p = 0.407). The baseline laboratory findings revealed that less IgG4-RD complicating malignancy patients had eosinophilia (5.9% versus 31.4%, p = 0.022) than controls.

### Related factors of malignancies in patients with IgG4-RD

Based on initial comparison results listed above, head and neck involvement, autoimmune pancreatitis and eosinophilia were related factors for having malignancies in IgG4-RD patients. As shown in Table 3, odds ratio were calculated by univariate analysis and we identified that head and neck involvement (OR 0.304, 95%CI 0.097–0.925, p = 0.041) and eosinophilia (OR 0.117, 95%CI 0.014–0.966, p = 0.046) were potentially negatively related to malignancies in patients with IgG4-RD and autoimmune pancreatitis (OR 5.359, 95%CI 1.651–17.939, p = 0.005) were positively related to malignancies in patients with IgG4-RD. Multivariate analysis confirmed that autoimmune pancreatitis (OR 6.230, 95%CI 1.559–24.907, p = 0.010) was an independent risk factor for IgG4-RD patients developing malignancies. In contrast, eosinophilia (OR 0.094, 95%CI 0.010–0.883, p = 0.039) was an independent protective factor from malignancy in patients with IgG4-RD.

**Table 3:** Related factors for malignancies in patients with IgG4-RD.

Variables	Univariate analysis		Multivariate analysis	
	Univariate OR (95%Cl)	P-value	Multivariate OR (95%Cl)	P-value
Head and Neck involvement	0.304(0.097–0.952)	0.041	0.604(0.152–2.401)	0.474
Autoimmune pancreatitis	5.359(1.651–17.393)	0.005	6.230(1.559–24.907)	0.010
Eosinophilia	0.117(0.014–0.966)	0.046	0.094(0.010–0.883)	0.039

### Treatment and prognosis

The mean initial dosage of corticosteroids (equivalent to prednisone) was 40.1 ± 10.0 mg/d in IgG4-RD complicating malignancy patients comparing to 32.4 ± 17.5 mg/d in controls. Among the seventeen IgG4-RD complicating malignancy patients, ten (58.8%) patients received immunosuppressive agents, including cyclophosphamide (2, 11.8%), mycophenolate mofetil (2, 11.8%), azathioprine (2, 11.8%), iguratimod (2, 11.8%) leflunomide (1, 5.9%) and methotrexate (1, 5.9%). Sixteen patients underwent resection therapy, with one lymphoma received chemotherapy. During a median follow-up period of 61.4 ± 26.4 months, all patients with IgG4-RD and malignancies survived. Two IgG4-RD patients without malignancy in the control group died due to liver failure and respiratory failure caused by other comorbidities.

## Discussion

To our knowledge, the present study is the first to report reliable data of Chinese IgG4-RD patients complicating malignancies and describes the largest cohort of IgG4-RD patients. We demonstrated malignancy incidence and distributions in IgG4-RD patients. Clinical characteristics of IgG4-RD patients with malignancy were compared, and potential risk and protective factors for IgG4-RD patients with malignancy were detected. We also summarized the prognosis of these patients.

The question of whether IgG4-RD patients are at an increased risk of malignancy has not been fully answered yet. As shown in Fig. 2, two studies from Japan reported no significant increase in the incidence of malignancy in IgG4-RD patients^7,8^. In contrast, other studies from Japan, the United States, the United Kingdom and South Korea^3–6,9,10^ have reported that IgG4-RD patients have a higher prevalence or incidence of malignancies. Similar to those studies, our study confirmed a total malignant SIR of 2.78 (95%CI 1.33–5.12) in Chinese patients.

When clinical physicians treat IgG4-RD patients, it is important to know what malignancy is most likely to occur. Our study reported that in Chinese IgG4-RD cohort, the most prevalent solid tumour was located in the gastrointestinal tract, followed by the thyroid, urinary system and breast. Different malignancy distributions were observed in studies from other countries, with the highest reported incidences of lung cancers^8^ gastric cancers^17^ and prostate cancers^5^, respectively, which may be due to geographic differences. Only one lymphoma case out of 17 malignancies were observed in our study, whereas a South Korean cohort reported 4 lymphoma cases (out of 12 malignancies)^3^.

Chinese IgG4-RD-CA patients have distinct clinical features. In this study, they were more prone to have autoimmune pancreatitis (AIP) and less likely to have head and neck gland involvement. Moreover, eosinophilia was less common in IgG4-RD-CA patients than in controls. Of note, other risk factors were reported including DM by Hirano’s group^8^ and IgG4-RD onset age >65 years^8^. This difference might be due to selection of controls, and the results need to be confirmed by larger and prolonged observational studies. Previous study detected that the presence of AIP was more closely associated with extra-pancreatic cancer than pancreatic cancer itself ^8,9,17–20^. And most cancer cases detected in AIP occur at or within a year of diagnosis^19^. These results are in line with our findings that AIP is a potential risk factor for complicating malignancies. K-ras gene mutations have been detected in the gastrointestinal tract in AIP patients, indicating pancreatic tissues of AIP patients might share similar immune responses with cancer^21^. Furthermore, cancer and pancreatic tissues of AIP patients with cancer share key immune responses leading to the enhancement of IgG4 antibody production^17^. These might contribute to the pathogenesis of AIP complicating with malignancies. Notably, we also found a potent independent protective indication for malignancy in IgG4-RD patients: eosinophilia. It was reported that a high proportion of patients have longstanding allergies, peripheral blood eosinophilia and serum IgE elevation and T helper type 2 responses was presumed to be pathogenic in IgG4-RD, which might indicate a close relationship between allergy and IgG4-RD^22^. Elevated serum IgE and peripheral eosinophilia may predict risk of flare after treatment, while a dominant eosinophilic infiltrate is not common in IgG4-RD involved organ^23^. In the present study, multivariate logistic regression analysis confirmed eosinophilia were significantly negative related to malignancy in IgG4-RD (OR 0.094, 95%CI 0.010–0.883, p = 0.039). This indicates the heterogenetic characteristics of IgG4-RD patients and subgroup analysis are needed^24^. No difference was observed between the serum IgG4 level between IgG4-RD patient with and without malignancies in this study. Prior findings demonstrated that mild elevation (within 1–2 upper limits of normal) of IgG4 has been observed in 5% to 15% of pancreatic carcinoma patients, and high levels of IgG4 were more specific for autoimmune pancreatitis (AIP)^25,26^. Controversially, Wallace^5^ and Asano^6^ reported that IgG4-RD patients with malignancy demonstrated a higher IgG4 serum level than IgG4-RD patients without malignancy. One possible explanation for the lower serum IgG4 levels in IgG4-RD-CA patients is that IgG4-RD, as a paraneoplastic syndrome, is able to induce limited elevations of IgG4 and atypical IgG4-related disease^6^. Additional studies evaluating the appropriate cut-off level of serum IgG4 concentrations affecting IgG4-RD prognosis and what kind of malignancies are more likely to accompany mild IgG4 elevation are required.

To our knowledge, no report has previously described the mortality and prognosis of IgG4-RD-CA patients. In the present study, during 61.4 ± 26.4 months of continuous follow-up, all IgG4 patients with malignancies survived. Although IgG4-RD patients had an increased incidence of malignance, the periodical follow-up and detailed examination of the recruited patients in our cohort might lead to an early detection and treatment of malignancy and improved the prognosis.

The pathophysiology of IgG4-RD complicating malignancy is still under investigation. It is well established that long-standing chronic inflammation plays a critical role in the development of cancer through the process of inflammation-associated carcinogenesis^27^. Our study showed that almost half of patients developed malignancies in the IgG4-RD background. Moreover, Asano et. al reported 12 patients experiencing malignancies in the same organ affected by IgG4-RD out of 27 IgG4-RD-CA patients^6^. It could be because IgG4-RD causes sustained inflammation that may induce preferable cancerous microenvironments, which resembles the mechanism of lymphoma development in association with Sjögren’s syndrome^28^. However, it has been reported that surgical resection of cancer was able to cause remission of IgG4-RD^25,29^, indicating that IgG4-RD might be a type of paraneoplastic syndrome^1^. Overall, it is still too early to draw a definitive conclusion of the pathogenesis of IgG4-RD and malignancies. Nevertheless, our study provides the critical information for physicians that IgG4-RD-affected organs should be carefully screened for potential malignancy occurrence during follow-up.

There are some limitations of this study. First, we only included consecutive patients from 2011 to 2018, and had not conducted an extensive long-term follow-up to the endpoint of death. Second, this is a single-center cohort study, and an increased number of patients should be continuously recruited. Third, this is a clinical observational study, and we did not investigate the detailed pathophysiology of these two disease entities, and further research in this field should receiving more attention.

In conclusion, this prospective cohort study demonstrated that Chinese IgG4-RD patients have a 2.78-fold increased risk of having malignancy over a median of 61.4 months of follow-up. The most common malignancy site was the gastrointestinal tract. Autoimmune pancreatitis was identified as a potential risk factor for IgG4-RD patients having malignancy, while eosinophilia as protective indicators.
